# Effect of music therapy on sleep quality in elderly: A systematic review and meta-analysis

**DOI:** 10.1371/journal.pone.0334356

**Published:** 2025-11-04

**Authors:** Cheng Li, Archana Prabu Kumar, Dapkupar Wankhar, Maheshkumar Kuppusamy, Karuppasamy Govindasamy

**Affiliations:** 1 School of Music and Dance, Ningxia Normal University, Xueyuan Road, Yuanzhou District, Guyuan, Ningxia, China; 2 Medical Education Department, College of Medicine and Health Sciences, Arabian Gulf University, Bahrain; 3 Faculty of Paramedical Sciences, Assam down town University, Guwahati, Assam, India; 4 Department of Physiology and Biochemistry, Govt.Yoga and Naturopathy Medical College and Hospital, Chennai, Tamilnadu; 5 Department of Sports, Recreation and Wellness, Symbiosis International (Deemed University), Hyderabad Campus, Modallaguda (V), Nandigama (M), Rangareddy, Hyderabad, Telangana, India; The Chinese University of Hong Kong, HONG KONG

## Abstract

**Background:**

Sleep disturbances are common among older adults, affecting up to 50% of this population and significantly impacting quality of life and health outcomes. Music therapy has been proposed as a non-pharmacological intervention to improve sleep quality in this population, but evidence regarding its effectiveness remains inconsistent across individual studies. Therefore, this systematic review and meta-analysis aims to synthesize available evidence on the effectiveness of music therapy interventions for improving sleep quality in elderly adults..

**Methods:**

We conducted a systematic review and meta-analysis following PRISMA guidelines. Major databases were searched for studies evaluating music interventions for sleep quality in adults aged 50 + years. Randomized controlled trials (RCTs) and non-randomized controlled trials (non-RCTs) were included. Risk of bias was assessed using the Cochrane Risk of Bias 2 (RoB 2) tool for RCTs and the Risk of Bias in Non-randomized Studies of Interventions (ROBINS-I) tool for non-RCTs. Standardized mean differences (SMDs) with 95% confidence intervals (CIs) were calculated using both common effect and random effects models. The certainty of evidence was evaluated using the Grading of Recommendations Assessment, Development and Evaluation (GRADE) approach.

**Results:**

The initial search retrieved 473 articles from electronic databases. After removing duplicates and screening, 10 studies (6 RCTs, 4 non-RCTs) published between 2010 and 2023 with 602 participants met inclusion criteria and were included in the meta-analysis. The random effects model showed significant improvement in sleep quality with music therapy (SMD: −0.79; 95% CI, −1.25 to −0.33; P < .001). Substantial heterogeneity was observed (I² = 79%; Q = 42.54; P < .001). Subgroup analysis revealed significant benefits in RCTs (SMD: −0.59; 95% CI, −1.11 to −0.07) but not in non-RCTs (SMD: −1.08; 95% CI, −2.36 to 0.19). Sensitivity analyses confirmed the robustness of findings, and no publication bias was detected. GRADE assessment indicated very low certainty of evidence for both RCTs and non-RCTs due to risk of bias concerns and substantial heterogeneity.

**Conclusions:**

Music therapy demonstrates significant improvement in sleep quality among older adults; however, the very low certainty of evidence based on GRADE assessment suggests caution in clinical recommendations. Future research should address methodological limitations, particularly regarding bias in outcome measurement and intervention implementation, to provide more definitive evidence for clinical practice guidelines.

## Introduction

Sleep is essential for good mental and physical health, with poor sleep quality negatively impacting cognition, emotion, and behavior regulation [1,2]. Sleep disturbance is particularly prevalent among older adults due to age-related changes in sleep architecture and circadian regulation [3]. According to epidemiological studies, 40–70% of older adults experience sleep problems, and up to 41.4% of community-dwelling older adults suffer from insomnia [4]. In the United States, 44.3% of elderly people report abnormal sleep, while in rural China, 33.8% of older adults experience poor sleep quality [5,6].

Common sleep complaints among older adults include “frequently waking up in the middle of the night” or “waking up early in the morning” [7]. These sleep disturbances can have severe consequences, including physical impairment, psychiatric illness (anxiety, depression, dementia, and suicidal behavior), and comorbidities such as hypertension, diabetes mellitus, immune disorders, and cardiovascular diseases [8,9]. Unresolved sleep problems in older adults lead to poor quality of life, cognitive impairment, emotional distress, decline in physical function, and increased risk of falling incidents [10].

Pharmacological treatment remains the primary approach for sleep disorders, particularly insomnia [11]. However, medications are only recommended for short-term use due to potential tolerance, dependence, and increased morbidity and mortality associated with long-term use. Elderly populations face additional medication-related risks including cognitive impairment, falls, and drug interactions with existing comorbidity treatments [12]. Therefore, exploring non-pharmacological, safe, and effective interventions to improve sleep quality in elderly people has become a worldwide concern [13].

Music therapy, based on ancient cross-cultural beliefs about music’s “healing” effect on mind and body, represents one such non-pharmacological intervention [14]. It is widely used as complementary treatment for psychological problems, cognitive impairment, pain, and sleep disturbances [15,16]. Music therapy can be active (involving singing or playing instruments) or passive (focusing on listening to music) [16]. Sedative music, characterized by a slow tempo of 60–80 beats per minute, soft volume, and smooth melody, may be particularly effective for improving sleep [17].

The mechanisms underlying music’s effects on sleep quality may involve several pathways. First, music is commonly applied to emotion self-regulation [18], potentially addressing the emotional disorders often associated with insomnia through effects on the brain’s limbic system [19]. Second, music influences physiological and psychological responses, enhancing parasympathetic activities by reducing plasma cytokine and catecholamine levels, decreasing cortisol levels, and lowering heart rate and respiratory rate [20]. In simpler terms, music helps activate the body’s natural relaxation response while reducing stress hormones and promoting restful physiological states. The psychophysiological theory underlying music therapy’s sleep-promoting effects involves multiple mechanisms. Music activates the parasympathetic nervous system through vagal stimulation, reducing cortisol levels while enhancing melatonin production to support natural circadian rhythms [20]. Musical stimulation modulates brainwave patterns toward relaxation-associated alpha and theta frequencies and triggers neurotransmitter release (serotonin, GABA) that facilitates sleep onset [21]. Additionally, music serves as an auditory distraction from intrusive thoughts and anxiety, while rhythmic entrainment with slow-tempo compositions (60–80 BPM) synchronizes biological rhythms with resting heart rates [22]. These neurobiological and psychological pathways collectively support music therapies potential as a non-pharmacological intervention for improving sleep quality in older adults.

Previous study on music interventions for sleep quality in older adults has yielded mixed results [23]. Some studies have confirmed their effectiveness [15,24,25], while others found no significant differences between intervention and control groups [26,27]. A Cochrane review concluded that music interventions effectively improve subjective sleep quality in adults with insomnia [28], and a network meta-analysis indicated clear advantages for adults with primary insomnia [29].However, existing systematic reviews have primarily focused on general adult populations with limited age-stratified analyses. Furthermore, previous studies examining elderly populations specifically have been characterized by small sample sizes, heterogeneous intervention protocols, and cultural bias toward Western populations, limiting the generalizability of findings to diverse elderly groups globally [28,29]. Although systematic reviews and meta-analyses have demonstrated the general effectiveness of music interventions for insomnia, comprehensive quantitative assessment of their impact specifically on elderly populations remains lacking. Older adults face unique sleep challenges including age-related circadian rhythm disruption, increased medication burden, and higher rates of comorbidities that may influence treatment response. This systematic review and meta-analysis aims to address this gap by evaluating the effects of music interventions on sleep quality in older adults.

## Methods

### Protocol and registration

This systematic review and meta-analysis was conducted according to the Preferred Reporting Items for Systematic Reviews and Meta-Analyses (PRISMA) guidelines (Suppl. File: S1 File).

### Search strategy

A comprehensive literature search was conducted in the following electronic databases: PubMed, Embase, Web of Science, Cochrane Library, CINAHL, PsycINFO, and China National Knowledge Infrastructure (CNKI). The search included articles published from database inception to August 2024. The following keywords and their combinations were used: “music,” “music therapy,” “music intervention,” “sleep,” “sleep quality,” “insomnia,” “elderly,” “older adults,” “aged,” and “geriatric.” Additionally, reference lists of included studies and relevant reviews were manually searched to identify further eligible studies.

### Eligibility criteria

Studies were included if they met the following criteria: (1) participants were adults aged 50 years or older; (2) investigated music interventions (active or passive) as the primary intervention; (3) included a control group receiving standard care, no intervention, or any intervention excluding pharmacological treatments and music therapy; (4) reported sleep quality outcomes; (5) were randomized controlled trials (RCTs) or non-randomized controlled trials (non-RCTs) including quasi-experimental, and pre-post studies. Studies were excluded if they: (1) included participants younger than 60 years; (2) used mixed interventions where the effect of music could not be isolated; (3) were duplicate publications or conference abstracts without full text; or (4) had insufficient data for effect size calculation.

### Study selection

Two independent (KG and DW) reviewers screened titles, abstracts, and keywords of all retrieved articles. Full texts of potentially eligible studies were then evaluated according to the inclusion and exclusion criteria. Any disagreements were resolved through discussion or consultation with a third reviewer (AMP).

### Data extraction

A standardized data extraction form was used to collect the following information: first author, publication year, country, study design, participant characteristics (sample size, age, gender, setting, health status), intervention details (type, duration, frequency, music characteristics), control group conditions, outcome measures, and results. Data extraction was performed independently by two reviewers (KG and CL), with discrepancies resolved through consensus.

### Risk of bias assessment

The risk of bias in RCTs was assessed using the Cochrane Risk of Bias 2 (RoB 2) tool, which evaluates five domains: randomization process, deviations from intended interventions, missing outcome data, measurement of outcomes, and selection of reported results [30]. For non-RCTs, the Risk of Bias in Non-randomized Studies of Interventions (ROBINS-I) tool was used, assessing seven domains: confounding, selection of participants, classification of interventions, deviations from intended interventions, missing data, measurement of outcomes, and selection of reported results [31]. Two reviewers independently conducted the assessments, with disagreements resolved through discussion.

### GRADE

The GRADE assessment was conducted by evaluating five key domains (risk of bias, inconsistency, indirectness, imprecision, and publication bias) at both outcome and study levels. Three authors independently assessed these domains, with each rated as neutral, serious, or very serious. Meta-analyses initiated with four points and were downgraded accordingly, resulting in final evidence ratings of High (4 points), Moderate (3 points), Low (2 points), or Very Low (≤1 point) [32].

### Data synthesis and statistical analysis

The primary outcome was sleep quality, primarily measured using the Pittsburgh Sleep Quality Index (PSQI), Stanford Sleepiness Scale (SSS), and electroencephalography (EEG) etc. Standardized mean differences (SMDs) with 95% confidence intervals (CIs) were calculated for continuous outcomes. Both common effect (fixed-effect) and random effects models were used to pool the results, with the random effects model considered primary due to anticipated heterogeneity. Heterogeneity was assessed using the I² statistic and Cochran’s Q test, with I² values of 25%, 50%, and 75% indicating low, moderate, and high heterogeneity, respectively. Prediction intervals were calculated to estimate the range of true effects in future studies. Subgroup analyses were performed based on leave-one-out sensitivity analyses were conducted to evaluate the robustness of findings and identify influential studies. Additional sensitivity analyses were planned to explore the impact of risk of bias on the results. Publication bias was assessed using funnel plots, Begg’s rank correlation test, and Egger’s linear regression test. A p-value <0.05 was considered statistically significant for all analyses. All statistical analyses were performed using R version 4.1.0 (R Foundation for Statistical Computing, Vienna, Austria) with the meta and metafor packages [33].

## Results

### Study selection

**Fig 1:**
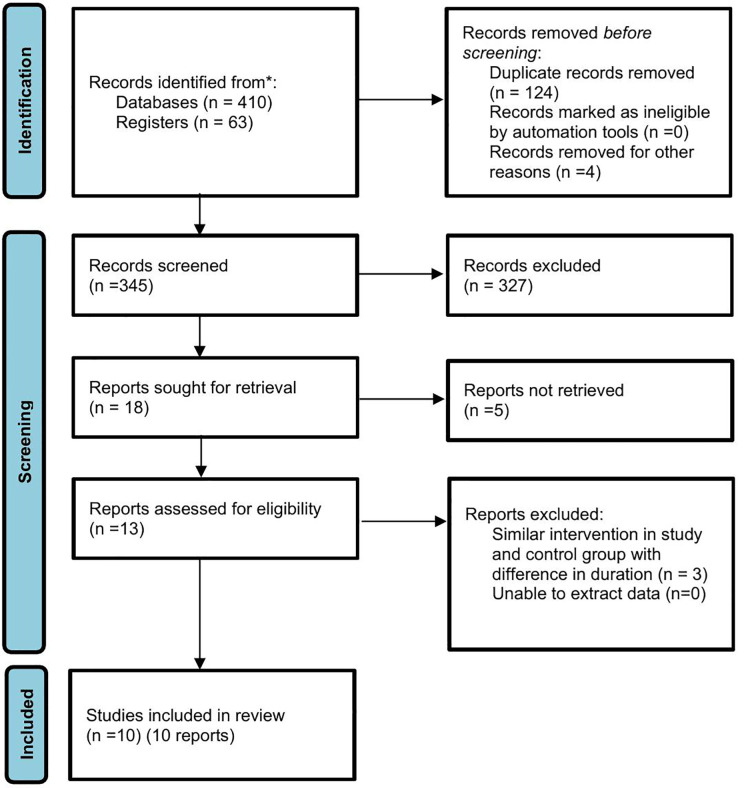
PRISMA 2020 flow diagram for systematic reviews which included searches of databases and registers.

The study selection process followed the PRISMA guidelines (Fig 1). The initial search retrieved 473 articles. After removing 128 duplicates, 345 articles underwent title, abstract, and keyword screening. Of these, 327 were excluded for not meeting study criteria, leaving 18 full-text articles for detailed evaluation. Ultimately, 10 studies published between 2010 and 2023 met all inclusion criteria and were included in the qualitative synthesis [24,26,27,34–40].

### Characteristics of the included studies

This meta-analysis included ten studies published between 2010 and 2023 investigating music interventions for sleep in older adults (Table 1). Study designs comprised randomized controlled trials (n = 6), [24,26,27,36,37,40] and non-randomized controlled trials (n = 4) [34,35,38,39]. Participants were predominantly older adults (aged 50 + years) with sample sizes ranging from 31 to 133, recruited from various settings including community centers, long-term care facilities, nursing homes, and hospitals. Several studies focused specifically on participants with poor sleep quality, while others included patients with specific medical conditions (hematologic cancer, post-surgical ICU patients). Interventions varied from passive music listening (predominant approach) to active music participation, with durations ranging from a single session to three months of regular application. Music types included classical (Western and Chinese), instrumental, nature sounds, religious music, and culturally-specific genres (e.g., Taiwanese Hokkien oldies with binaural beats). Most interventions utilized slow tempo music (60–85 BPM) with session durations of 20–60 minutes, applied with frequencies varying from daily to weekly. Sleep outcomes were primarily measured using the Pittsburgh Sleep Quality Index (PSQI), with some studies incorporating additional measures such as polysomnography, visual analog scales, or biochemical markers.

**Table 1:** Characteristics of included studies on music interventions for sleep in older adults.

Study	Country	Design	Participants	Sample Size (Mean Age)	Intervention Description	Music Type & Characteristics	Duration & Frequency	Control Group	Sleep Measures	Key Sleep Outcomes
Chan (2010)	Hong Kong	RCT	Healthy older adults (60+)	N = 42 (IG: 21, CG: 21) Age: 75+	MP3 player listening + relaxation instructions	Meditative, Chinese classical, Western classical, jazz Slow, flowing pieces60–80 BPM	30 min daily × 4 weeks	Uninterrupted rest period	PSQI	No significant differences between groups
Shum (2014)	Singapore	RCT	Older adults with poor sleep	N = 60 (IG: 28, CG: 32) Age: 55+	MP4 music player with earphones + relaxation instructions	Western classical (Bach, Mozart, Chopin), Chinese classical, jazz, New Age Soft, instrumental, slow 60–80 BPM, no lyrics	40 min weekly × 6 weeks	No music listening for 6 weeks	PSQI	PSQI scores decreased in IG while CG remained unchanged
Wang (2016)	China	RCT	Older adults with poor sleep	N = 64 (IG: 32, CG: 32) Age: 69.4 ± 5.5 years	MP3 player listening + relaxation instructions	Chinese instrumental classical, Western classical, natural sounds, classical songs Soft, sedative, stable melodies60–80 BPM, no lyrics	30-45 min nightly × 3 months	Sleep hygiene education	PSQI	Greater improvements in global PSQI score at each time point vs. control
Lai (2015)	Taiwan	Cross-over RCT	Older adults with insomnia	N = 38 Age: 59.6 ± 6.7 years	Music videos with nature scenes + Buddhist teachings	Peaceful music with minor tonalities and smooth melodies60–85 BPM	30 min × 1 night	Usual care	PSG: TST, SE, SOL, WASO, awakenings, sleep stages, arousal index	Significantly shorter SOL in music video condition (p = .002)
Yap (2017)	Singapore	RCT	Older adults	N = 31 (IG: 16, CG: 15) Age: 74.7 ± 6.4 years	Group Rhythm Wellness Programme with percussion instruments	Rhythmic music using percussion instruments (conga, cowbell, djembe, ashiko, dunum, shakers, wood blocks)	1 hour weekly × 10 weeks	Usual care	PSQI	No significant differences in PSQI scores between groups
Kubra & Mine (2020)	Turkey	Experimental pre-post control design	Older hematologic cancer patients	N = 54 (Exp: 27, Control: 27) Age: 65+	Daily music listening	Hejaz, Husseini, and Neva compositions Nonverbal instrumental music from TUMATA health series	Daily × 1 week	Routine care without music	PSQI, STAI	Significant improvement in sleep quality (PSQI) in experimental group
Altan (2016)	Turkey	Pre-post design	Nursing home residents	N = 31 Age: 81.0 ± 8.49 years	Passive music therapy at bedtime with other residents	Ussak Maqam music Tempo not specified Not participant-selected	60 min at bedtime, 7 times/week × 3 weeks	None (pre-post design)	PSQI	Positive effects on sleep quality
Sun (2015)	China	Non-RCT	Community-dwelling older adults	N = 100IG Age: 67.16 ± 3.6 years	Active group music therapy: discussing, creating, relaxation	Music database and music creation Tempo not specified Not participant-selected	4 times/week × 8 weeks (Duration uncertain)	Four sleep promotion education sessions	PSQI	Improved sleep quality except sleep medication component
Guo (2019)	China	Non-RCT	Older adults with sleep difficulties (PSQI > 7)	N = 65IG Age: 85.44 ± 3.78 years	Passive music therapy: listening, discussing, recalling	Five-element music Tempo not specified Not participant-selected	30-60 min, 7 times/week × 8 weeks	Routine pension agency care	PSQI	Improved sleep quality, especially in sleep quality, latency, duration, disturbance, and medication components
Lin (2023)	Taiwan	Single-blind RCT	Long-term care institution residents	N = 64 (32 per group) BBM: 81.72 ± 2.83Control: 82.06 ± 3.68	Taiwanese Hokkien oldies with embedded binaural beats **Morning:** 25 Hz (β frequency) **Afternoon:** 14 → 5 Hz (α → θ frequency)	Taiwanese Hokkien oldies Familiar music selected by participants Binaural beat frequencies embedded	20 min, 3 times/week (Mon, Wed, Fri) × 2 weeks (12 sessions total)	Same music without binaural beats	PSQI, HRV	Significant decrease in habitual sleep efficiency (PSQI)

### ROB assessment

**Fig 2:**
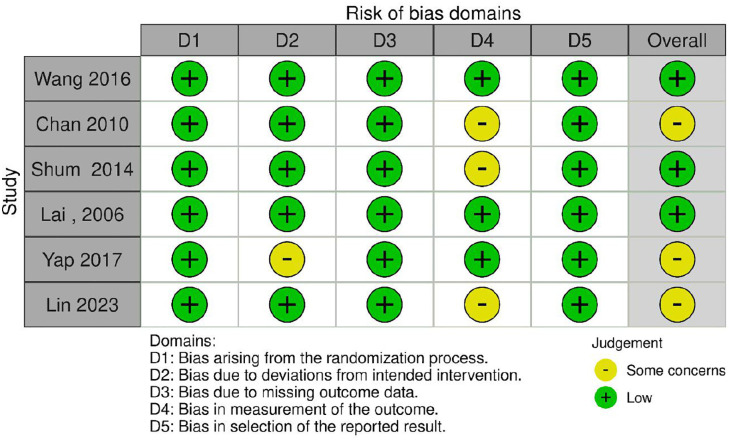
Risk of bias for RCTs studies included.

**Fig 3:**
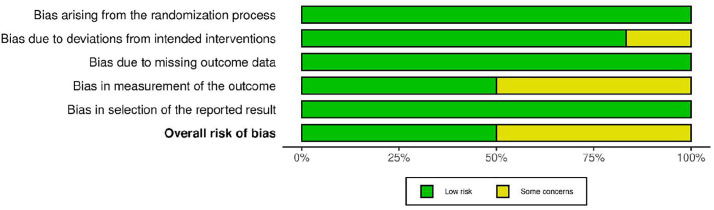
Risk of Bias summary for RCTs included.

Among the six RCTs (Fig 2 and 3), four studies [24,26,36,37] demonstrated low risk of bias across most domains, while two studies [27,40] had some concerns regarding measurement of outcomes and deviations from intended interventions, respectively.

**Fig 4:**
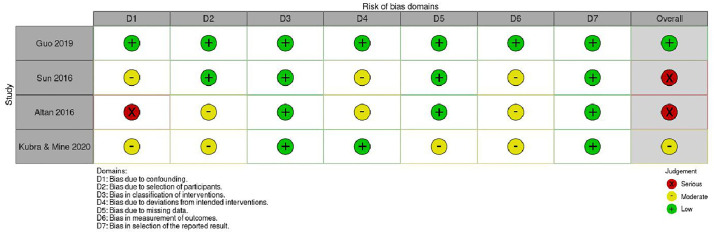
Risk of Bias for Non RCTs using ROBINS-I tool.

**Fig 5:**
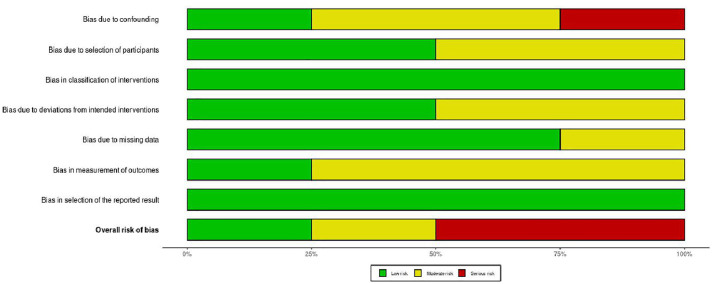
Risk of Bias with summary for Non RCTs.

For the four non-RCTs (Fig 4 and 5), one study [35] showed low risk of bias across all domains, one study [34] demonstrated moderate risk of bias primarily due to issues with participant selection and outcome measurement, and two studies [38,39] were assessed as having serious risk of bias due to confounding factors and measurement concerns.

### Meta analysis

**Fig 6:**
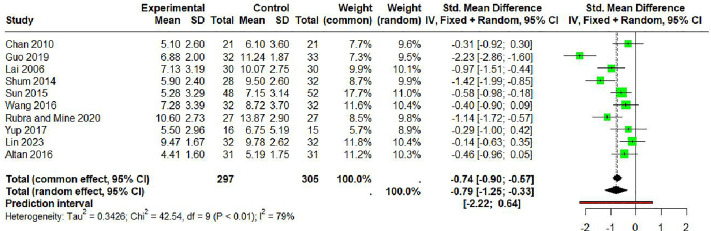
Forest plot for PSQI with music therapy in elderly.

Ten studies comprising 602 participants (297 in intervention groups, 305 in control groups) were included in the meta-analysis. The random effects model (Fig 6) showed a significant improvement in sleep quality with music therapy (SMD: −0.79; 95% CI, −1.25 to −0.33; P < 0.001). Substantial heterogeneity was observed among studies (I² = 79%; Q = 42.54, df = 9; P < 0.001). The prediction interval (−2.22 to 0.64) indicated considerable variability in treatment effects across settings.

### Subgroup analysis

**Fig 7:**
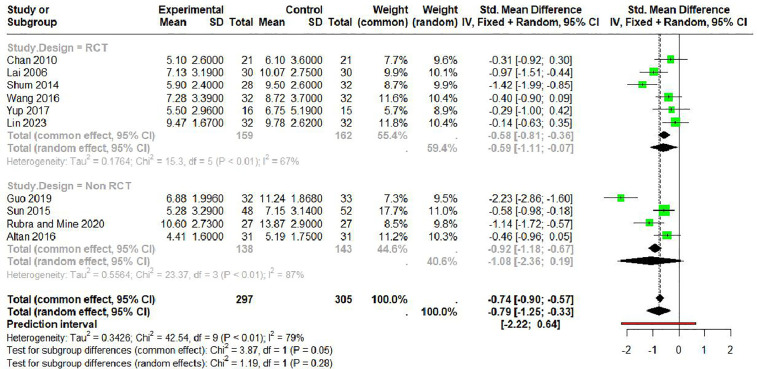
Forest plot for PSQI by study design with music therapy in elderly.

We performed subgroup analyses based on study design (Fig 7). For RCTs (k = 6), the random effects model showed a significant improvement in sleep quality (SMD: −0.59; 95% CI, −1.11 to −0.07; I² = 67%), while non-RCT studies (k = 4) showed no statistically significant effect (SMD: −1.08; 95% CI, −2.36 to 0.19; I² = 87%). The test for subgroup differences in the random effects model was not significant (Chi² = 1.19, df = 1, P = 0.28). Moderate to substantial heterogeneity was observed within both RCT and non-RCT subgroups, with non-RCTs showing higher heterogeneity than RCTs.

### Meta regression

Meta-regression analyses were conducted to explore sources of heterogeneity, examining intervention characteristics including duration, session frequency, total sessions, delivery method, and music type. Among the successfully analyzed moderators, study design explained minimal variance (R² = 2.97%, QM = 1.41, p = 0.235), while intervention duration (β = 0.0009, p = 0.987), session duration (β = −0.0052, p = 0.722), frequency per week (β = −0.0171, p = 0.833), and total sessions (β = −0.0017, p = 0.847) showed no significant associations with effect size. The limited explanatory power of these moderators (R² range: 0–2.97%) suggests that unmeasured factors, such as participant characteristics, outcome measurement methods, or intervention fidelity, may contribute substantially to the observed heterogeneity between studies.

### Sensitivity analysis

**Fig 8:**
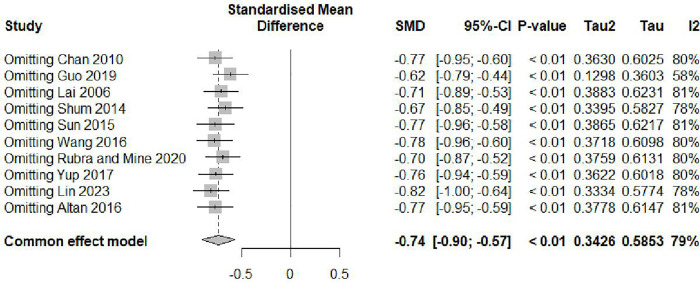
Leave-one-out sensitivity analysis forest plot.

Leave-one-out sensitivity analysis demonstrated robustness of findings (Fig 8), with all iterations maintaining statistical significance in the common effect model (all P < 0.01). The SMD values ranged from −0.62 (omitting Guo 2019) to −0.82 (omitting Lin 2023), with the overall pooled estimate being −0.74 (95% CI, −0.90 to −0.57). Heterogeneity remained substantial across all iterations, with I² values ranging from 78% to 81% and Tau² values from 0.3334 to 0.3883. The Tau values were consistently around 0.58–0.63 across all analyses. Despite these variations, the direction and significance of the effect remained consistent across all sensitivity analyses, suggesting the findings were not dependent on any single study.

### Publication Bias Assessment

**Fig 9:**
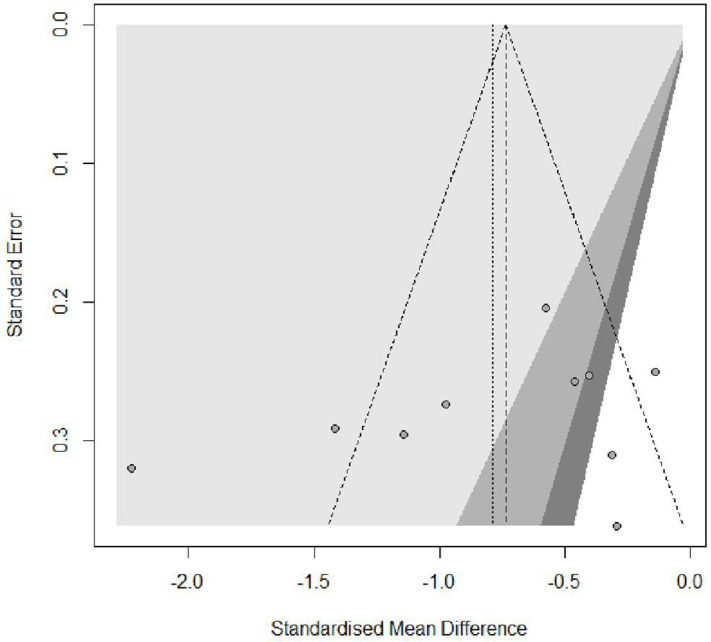
Funnel plot.

Tests for publication bias showed no significant asymmetry using Begg’s rank correlation (z = 0.27, P = .78) or Egger’s linear regression methods (t = −0.03, P = .97). The funnel plot (Fig 9) suggest that publication bias did not substantially influence the observed results in this meta-analysis of music therapy effects on sleep quality in the elderly.

### Summary of evidence with GRADE

Based on the GRADE assessment (Table 2), there is very low reliability of evidence for the effect of music therapy on sleep quality in elderly populations for both RCTs and non-RCTs. For RCTs, the evidence was downgraded by one level due to risk of bias concerns (three RCTs - Chan 2010, Shum 2014, and Lin 2023 showed unclear bias in outcome measurement [24,27,37], while Yap 2017 showed some concerns in deviation from the intended intervention) [40] and further downgraded by two levels due to high heterogeneity across studies. For non-randomized studies, the evidence was downgraded by two levels due to serious risk of bias in two studies (Sun 2016 and Alten 2016) [38,39] and by an additional two levels due to substantial heterogeneity.

**Table 2:** GRADE summary for the included studies.

Outcomes	Anticipated absolute effects^*^ (95% CI)		Relative effect (95% CI)	№ of participants (studies)	Certainty of the evidence (GRADE)	Comments
	Risk with Control	Risk with Music Therapy				
Sleep quality index	–	SMD 0.59 SD lower (1.11 lower to 0.07 lower)	–	321 (6 RCTs)	⨁○○○ Very low^a,b^	Music therapy may increase/have little to no effect on sleep quality index but the evidence is very uncertain.
Sleep Quality index	–	SMD 1.08 SD lower (2.36 lower to 0.19 higher)	–	281 (4 non-randomised studies)	⨁○○○ Very low^b,c^	Music Therapy may increase/have little to no effect on sleep Quality index but the evidence is very uncertain.

*The risk in the intervention group (and its 95% confidence interval) is based on the assumed risk in the comparison group and the **relative effect** of the intervention (and its 95% CI).

**CI** confidence interval; **SMD:** standardised mean difference

## Discussion

In this systematic review and meta-analysis of 10 studies involving 602 participants, we found evidence suggesting music interventions may improve sleep quality in older adults. The random effects model demonstrated a significant improvement (SMD: −0.79;), indicating a moderate to large effect size. However, substantial heterogeneity was observed among studies (, with a wide prediction interval (−2.22 to 0.64) indicating considerable variability in treatment effects across different settings and populations.

Our results align with previous meta-analyses examining music therapy for sleep quality in older adults. Chen et al. found that music significantly improved sleep quality in older adults (mean difference: −1.96; 95% CI, −2.23 to −1.73; P = .003), with greater benefits from sedative music and interventions lasting longer than 4 weeks [41]. Similarly, Wang et al. reported significant improvements in sleep quality across 9 studies (mean difference: −2.64; 95% CI, −3.76 to −1.53; P < .001), despite substantial heterogeneity (I² = 75.0%) [42]. In contrast, Petrovsky et al. found mixed evidence regarding efficacy, emphasizing the importance of personalized music selection [43].

Our subgroup analysis revealed important differences between study designs. RCTs (k = 6) demonstrated a significant improvement in sleep quality (SMD: −0.59; 95% CI, −1.11 to −0.07; I² = 67%), while non-RCT studies (k = 4) showed no statistically significant effect (SMD: −1.08; 95% CI, −2.36 to 0.19; I² = 87%). Although the test for subgroup differences was not significant (Chi² = 1.19, df = 1, P = 0.28), the higher heterogeneity observed in non-RCTs suggests greater methodological variability and potential bias. The risk of bias assessment supports this interpretation, as two of the four non-RCTs were assessed as having serious risk of bias due to confounding factors and measurement concerns, while five of the six RCTs demonstrated low risk of bias across most domains.

Importantly, our GRADE assessment indicated very low certainty of evidence for both RCTs and non-RCTs. For RCTs, evidence was downgraded by one level due to risk of bias concerns (three RCTs showed unclear bias in outcome measurement, and one showed concerns in deviation from intended interventions) and by two levels due to high heterogeneity across studies. For non-RCTs, evidence was downgraded by two levels due to serious risk of bias in two studies and by an additional two levels due to substantial heterogeneity. This very low certainty rating significantly limits our confidence in the estimated effects and suggests that future research may substantially change our understanding of music therapy’s effectiveness for sleep in older adults.

Meta-regression analyses explored potential sources of the substantial heterogeneity observed. However, intervention characteristics including duration, session frequency, total sessions, delivery method, and music type showed minimal explanatory power (R² range: 0–2.97%). This suggests that unmeasured factors, such as participant characteristics, outcome measurement methods, or intervention fidelity, may contribute substantially to the observed heterogeneity between studies.

A notable limitation across the included studies was the predominant reliance on subjective sleep quality measures, particularly the PSQI, rather than objective sleep assessment tools such as polysomnography or actigraphy. While the PSQI is a validated and widely-used instrument for assessing subjective sleep quality, it is susceptible to recall bias, social desirability bias, and may not accurately reflect objective sleep parameters such as sleep latency, sleep efficiency, or sleep architecture. The reliance on subjective measures may have contributed to the observed heterogeneity and limits our ability to determine whether music therapy produces measurable physiological changes in sleep patterns. The limited ability to explain heterogeneity through measured moderators highlights the complexity of music therapy interventions and the need for more standardized approaches in future research.

Sensitivity analyses demonstrated the robustness of our findings, with all leave-one-out iterations maintaining statistical significance and SMD values ranging from −0.62 to −0.82. Despite variations in individual study contributions, the direction and magnitude of the overall effect remained consistent, suggesting that our findings were not dependent on any single study. Publication bias assessment showed no significant asymmetry using both Begg’s rank correlation (z = 0.27, P = .78) and Egger’s linear regression methods (t = −0.03, P = .97), indicating that publication bias did not substantially influence our results.

Several mechanisms may explain music interventions potential benefits for sleep. Music can influence emotional regulation, potentially addressing emotional disturbances associated with sleep disorders in older adults [44,45]. Physiologically, music has been shown to reduce cortisol levels, heart rate, and respiratory rate, promoting relaxation and facilitating sleep initiation [46]. Music may also help form new psychological associations with bedtime, replacing maladaptive habits that interfere with sleep [47].

Our findings suggest music interventions represent a promising non-pharmacological approach for improving sleep quality in older adults, particularly given concerns with long-term pharmacological treatments. However, substantial heterogeneity and the wide prediction interval (−2.22 to 0.64) indicate individual responses vary considerably, highlighting the need for personalized approaches. Most interventions utilized slow tempo music (60–85 BPM), consistent with sedative music recommendations, though considerable variation existed in intervention characteristics across studies.

### Future research directions

Future research should develop standardized protocols including specific guidance on music selection, session duration, and delivery methods. Studies should employ rigorous methodological approaches with adequate randomization, blinding where possible, and standardized outcome measures. Importantly, future studies should incorporate objective sleep assessment methods (polysomnography or actigraphy) alongside subjective measures to determine whether improvements correspond to measurable physiological changes.

Research should investigate moderators of treatment effectiveness, including participant characteristics (baseline sleep quality, comorbidities, musical preferences) and intervention parameters (music type, duration, timing). Longer follow-up periods and detailed reporting of intervention characteristics would help identify optimal approaches for personalized interventions.

### Strengths and limitations

Strengths include comprehensive search strategies, rigorous methodological assessment, thorough statistical analyses with both effect models, and systematic GRADE evaluation. The absence of publication bias enhances reliability of findings.

Key limitations include substantial heterogeneity limiting definitive conclusions, relatively small number of studies (particularly non-RCTs), diversity of intervention characteristics precluding detailed moderator analyses, and very low certainty of evidence requiring cautious interpretation.

## Conclusions

Music interventions show promise for improving sleep quality in older adults, with our meta-analysis demonstrating significant effects. However, substantial heterogeneity and very low certainty of evidence require cautious interpretation. Music therapy represents a potentially safe, non-invasive approach for sleep disturbances in elderly populations, but high-quality randomized controlled trials with standardized interventions are needed to establish definitive effectiveness and optimal implementation strategies.

## Supporting information

S1 File PRISMA Checklist. (DOCX)

S2 File Master data. (PDF)
