# Supplementary material for: Effect of music therapy on sleep quality in elderly: A systematic review and meta-analysis
Source: PLoS One. 2025 Nov 4;20(11):e0334356. doi: 10.1371/journal.pone.0334356 (PMC12585030; doi:10.1371/journal.pone.0334356)
Supplement: S2 File — (PDF) [file pone.0334356.s002.pdf]

Author	Me	Se	Ne	Mc	Sc	Nc	Study desig	Duration (v	Session (m
Chan 2010	5.1	2.6	21	6.1	3.6	21	RCT	>3 weeks	?30 min
Guo 2019	6.88	1.996	32	11.24	1.868	33	Non RCT	>3 weeks	> 30 min
Lai 2006	7.13	3.19	30	10.07	2.75	30	RCT	? 3 weeks	?30 min
Shum 2014	5.9	2.4	28	9.5	2.6	32	RCT	>3 weeks	> 30 min
Sun 2015	5.28	3.29	48	7.15	3.14	52	Non RCT	>3 weeks	> 30 min
Wang 2016	7.28	3.39	32	8.72	3.7	32	RCT	>3 weeks	> 30 min
Kübra and	10.6	2.73	27	13.87	2.9	27	Non RCT	? 3 weeks	> 30 min
Yup 2017	5.5	2.96	16	6.75	5.19	15	RCT	>3 weeks	> 30 min
Lin 2023	9.47	1.67	32	9.78	2.62	32	RCT	? 3 weeks	?30 min
Altan 2016	4.41	1.6	31	5.19	1.75	31	Non RCT	? 3 weeks	> 30 min

Frequency,	Total Sessi	Music Type	Delivery M	Population
> 3 times	> 10 sessio	Mixed clas	Individual l	Healthy older adults
> 3 times	> 10 sessio	Five-eleme	Group pass	Sleep difficulties
?3 times	?10 sessior	Music with	Individual v	Insomnia
?3 times	?10 sessior	Mixed clas	Individual l	Poor sleep
> 3 times	> 10 sessio	Active mus	Group acti	Community dwelling
> 3 times	> 10 sessio	Mixed clas	Individual l	Poor sleep
> 3 times	?10 sessior	Turkish cla	Individual l	Cancer patients
?3 times	?10 sessior	Percussion	Group acti	Older adults
?3 times	> 10 sessio	Binaural be	Individual (	LTC residents
> 3 times	> 10 sessio	Ussak Maq	Group pass	Nursing home
